# Hospitalization due to drug use did not change after a decade of the Psychiatric Reform

**DOI:** 10.1590/S1518-8787.2016050006085

**Published:** 2016-05-06

**Authors:** Alexandre Dido Balbinot, Rogério Lessa Horta, Juvenal Soares Dias da Costa, Renata Brasil Araújo, Simone Poletto, Marina Bressaneli Teixeira

**Affiliations:** I Programa de Pós-Graduação em Saúde Coletiva. Universidade do Vale do Rio dos Sinos. São Leopoldo, RS, Brasil; II Programas de Dependência Química e Terapia Cognitivo-Comportamental. Hospital Psiquiátrico São Pedro. Porto Alegre, RS, Brasil; III Curso de Psicologia. Universidade do Vale do Rio dos Sinos. São Leopoldo, RS, Brasil; IV Residência Médica em Pediatria. Universidade Federal de Ciências da Saúde de Porto Alegre. Porto Alegre, RS, Brasil

**Keywords:** Substance-Related Disorders, Mental Disorders, Hospitals, Psychiatric, utilization, Hospitalization, Length of Stay, Time Series Studies

## Abstract

**OBJECTIVE:**

To investigate whether the psychiatric hospitalization rates due to use of psychoactive substances and average time of hospitalization suffered any changes after the first decade of effective implementation of the psychiatric reform in Brazil.

**METHODS:**

This article examines the evolution of hospitalizations due to disorders arising from the use of alcohol or other substances in the state of Santa Catarina, Southern Brazil, from 2000 to 2012. This is an ecological, time-series study, which uses data from admissions obtained by the Informatics Service of the Brazilian Unified Health System. Hospitalization rates by 100,000 inhabitants and average time of occupancy of beds were estimated. Coefficients of variation of these rates were estimated by Poisson Regression.

**RESULTS:**

The total and male hospitalization rates did not vary (p = 0.056 and p = 0.244, respectively). We observed an increase of 3.0% for the female sex (p = 0.049). We did not observe any significant variation for occupancy time of beds.

**CONCLUSIONS:**

The deployment of services triggered by the Brazilian psychiatric reform was not accompanied by a reduction of hospitalization rates or mean occupancy time of hospitalized patients during this first decade of implementation of the reform.

## RESUMO

**OBJETIVO:**

Investigar se as taxas de internações psiquiátricas por uso de substâncias psicoativas e o tempo médio de hospitalização se modificaram após a primeira década de efetiva implementação da reforma psiquiátrica no Brasil.

**MÉTODOS:**

Este artigo analisa a evolução das hospitalizações devido a transtornos decorrentes do uso de álcool ou outras substâncias no estado de Santa Catarina, de 2000 a 2012. Trata-se de estudo ecológico, de série temporal, com dados de internações obtidas do Serviço de Informática do Sistema Único de Saúde. Foram estimadas taxas de hospitalização por 100 mil habitantes e tempo médio de ocupação dos leitos. Foram estimados coeficientes de variação dessas taxas por Regressão de Poisson.

**RESULTADOS:**

As taxas de internação total e para o sexo masculino não variaram (p = 0,244 e p = 0,056, respectivamente). Houve incremento de 3,0% para o sexo feminino (p = 0,049). Não se observou variação significativa para tempo de ocupação dos leitos.

**CONCLUSÕES:**

A implantação de serviços desencadeada pela reforma psiquiátrica brasileira não foi acompanhada de redução das taxas de hospitalização ou tempo médio de permanência de quem foi hospitalizado ao longo dessa primeira década de implantação da reforma.

## INTRODUCTION

Mental health care in Brazil suffered a strong change in its paradigm in the recent decades as a result of the psychiatric reform law. That law had as its core the search for deinstitutionalization of people with psychic suffering, characterized, mainly, for what was called “anti-asylum fight”^15^. The previous model, hospital-centered, was abandoned and the network center of attention began to be care using substitute services, or centers of psychosocial care (CAPS)^15^ ^,^ ^a^ ^,^ ^b^.

This change takes place from the consolidation of the rights of individuals suffering from psychic distress, in 2001, by means of Law no. 10,216. It highlighted that psychiatric hospitalization is only recommended when treatment in services outside of hospitals may be insufficient, and it must happen in general hospitals^4^ ^,^ ^c^. One of the main features for achieving change in care was restricting the increase of beds in psychiatric hospitals, directing the public investment to the implementation of substitute services in the municipalities^10^. This should reduce the need and, at the same time, the number of hospitalizations^4^. The substitutive service of CAPS is marked by the ethical commitment with the right to a decent life, in spite of mental illness or other social and economic limitations. Still, it articulates the psychosocial care network, which includes all other services^b^. Psychosocial care networks can have five different settings, according to the size of the municipality. One of them is articulated by CAPS-AD (CAPS alcohol and other drugs)^a^ ^,^ ^b^.

In addition, in 2003, the Country adopted the National Policy Specific to Alcohol and other Drugs. This policy, along with the policy of the Ministry of Health for Integral Care to Users of Alcohol and other Drugs (2004), reinforces the guideline of giving priority to CAPS, and in particular to CAPS-AD, when available in the territory^d^. The CAPS-AD must offer daily care to the users, as well as management of cases, with personalized care, conditions for home and ambulatory detoxification for users who require it, care for family and prevention of relapses^3^, in addition to articulate the network with the basic services, clinics and specialized and reference hospital units^d^.

While this has been structured, population studies detect expansion of consumption of psychoactive substances, with increasing prevalence of use in life among men (77.0% to 81.7%) and women (from 62.5% to 68.3%) in the South region of Brazil^e^. Specifically for use in the life of crack, prevalence of use in the year of 2000 had not been reported, while in 2005 the prevalence reached 1.1% of the population (of both sexes), being 2.2% among men^e^. Among middle and high school students from public and private schools, in the state of Santa Catarina, Brazil, the consumption of psychotropic substances has increased significantly from 2004 (18.4%) to 2010 (35.7%)^f^.

There is no evidence, so far, that the implementation of the new model can, in fact, reduce the occupation of hospital beds. Hospitalization appears to keep being used as one of the main tools for promoting abstinence in initial periods of treatment. The hospitalizations are accounted for by the Hospital Information System (HIS) of the Brazilian Unified Health System (SUS), via requests for Authorization for Hospitalization (AIH), and made available by the computer Service of the unified health system, the DATASUS^4^.

This study investigated whether the psychiatric hospitalization rates due to use of psychoactive substances and average time of hospitalization suffered any changes after the first decade of effective implementation of the psychiatric reform in Brazil.

## METHODS

It is an ecological study, using a historical series consisting of secondary data provided by DATASUS. The data were collected directly from the system during the month of November, 2013. As collection strategy, we accessed a health information database (Tabnet), within the “epidemiological and morbidity” session, checking the “SUS hospital morbidity” option, according to place of residence (state of Santa Catarina, Southern Brazil).

The data were filtered using the list of morbidities of ICD-10^g^, selecting only the diagnoses among F10 (mental and behavioral disorders due to use of alcohol) and F19 (mental and behavioral disorders due to use of other psychoactive substances).

Individuals aged 15 years or more were included in the study. Data on people aged less than 15 years and those characterized by DATASUS as of “unknown age” were excluded.

Two dependent variables were created:

- hospitalizations due to diagnoses of mental and behavioral disorders due to use of alcohol and other psychoactive substances (ICD-10: F10-F19);
- average time of hospitalization due to diagnoses of mental and behavioral disorders due to use of alcohol and other psychoactive substances.

Population data needed for calculation of the hospitalization rates were also available on the website of the DATASUS, according to sex. The rates were calculated by the following equation: hospitalization rate = (total hospitalizations per group of causes by sex in the year)/(total population by sex in the year) x 100,000 inhabitants.

DATASUS data were exported to spreadsheets and analyzed in the Stata 11.1 program.

The specificities of gender for the occupation of beds in hospitals and health services justified the stratification according to sex in this analysis^18^. The coefficients according to the sex of the hospitalized patient were analyzed by Poisson Regression, with robust variance, their 95% confidence intervals and statistical Wald test. To assess the adequacy of the analyzed model, we used the χ^2^ test, Goodness-of-fit, determining as appropriate value an adjustment of p > 0.05^1^. The Poisson Regression coefficient showed the variation of hospitalization rates and average times of hospitalization, according to sex, over the period.

As those are secondary data provided by the Informatics Service of SUS, without identifying the individual who gave rise to this data, and since this information is in the public domain and available to the whole population, this study did not require evaluation by a Research Ethics Committee.

## RESULTS

**Figure 1:**
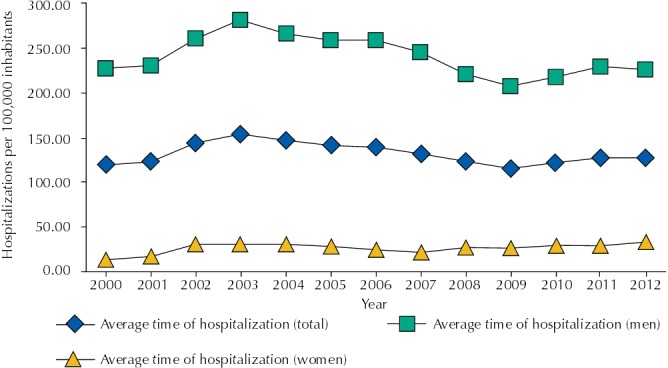
Psychiatric hospitalization rates due to use of alcohol and other drugs by 100,000 inhabitants, according to sex. Santa Catarina, Southern Brazil, 2000-2012.

We analyzed 76,696 hospitalizations recorded in the state of Santa Catarina, 68,647 being of male individuals. We observed an average of 132.64 (SD = 11.96) hospitalizations per 100,000 inhabitants per year, being the minimum rate 116.19 per 100,000 inhabitants in 2009, and the maximum 154.67 per 100,000 inhabitants in 2003. For females, the average of hospitalization was 27.12 per 100,000 inhabitants (SD = 5.29) per year and; for males, the average was 241.16 by 100,000 inhabitants (SD = 22.45) per year. The evolution of hospitalization rates is presented in Figure 1.

**Figure 2:**
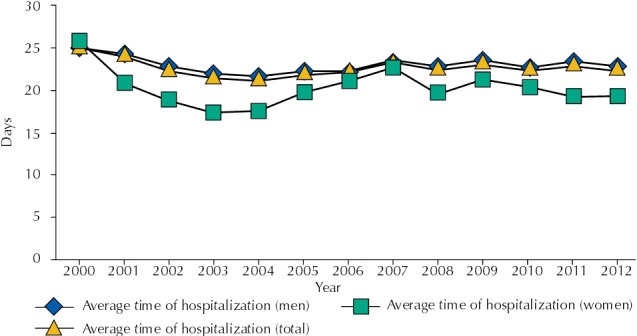
Average time of occupancy of hospital beds, in number of days, as a result of the use of alcohol and other drugs, according to sex. Santa Catarina, Southern Brazil, 2000-2012.

The average time of occupancy of hospital beds for the total population was of 22.55 days (SD = 1.04), the minimum being 21.1 days in 2004 and the maximum 24.9 days in 2000. By stratifying the analysis for the sexes, we observed a significant difference between the averages, with 20.21 days (SD = 2.27) for females and 22.87 days (SD = 0.95) for males (p < 0.001). The evolution of times of occupancy of hospital beds during hospitalization is presented in Figure 2.

Over the period studied, we did not observe significant variation in the hospitalization rates for both sexes (p = 0.244) or for male patients (p = 0.056). However, for female patients we estimated an increase of 3.0% in the hospitalization rates for 100,000 inhabitants per year in the period (p = 0.049) (Table).

**Table:** Poisson Regression coefficients for hospitalization rates and average time of occupancy of beds. Santa Catarina, Southern Brazil, 2000-2012.

Variable	Sex	Coefficient	95%CI	p
Hospitalization rate by 100,000 inhabitants/year	Total	0.992	0.979–1.005	0.244
	Male	0.998	0.976–1.000	0.056
	Female	1.029	1.000–1.059	0.049
Average time of occupancy of hospital beds	Total	0.997	0.990–1.004	0.411
	Male	0.997	0.991–1.004	0.450
	Female	0.994	0.976–1.012	0.520

Regarding the time of occupancy of hospital beds, we did not observe any significant variation for any of the categories analyzed in this study: male sex (p = 0.450), female sex (p = 0.520), and total (p = 0.411) (Table).

## DISCUSSION

The indicators examined show that the state of Santa Catarina has been following the tradition of greater occupation by men than by women of beds for the treatment of psychiatric disorders arising from the use of alcohol and other drugs. The first studies that analyzed this phenomenon suggested that among men, these were public domain behaviors and, for women, they were private behavior^15^. The less public visibility of these behaviors may have contributed to the reduced offer of beds and places for the confrontation of the problems arising from the consumption of alcohol and other drugs among women, over time. The reduced supply of attention in health services, in turn, reinforces the invisibility of these behaviors, except when accompanied by significant developments, such as psychotic symptoms. These conditions seem to be interconnected.

Prevalence studies of use of drugs point out consumption growth of alcohol and other drugs among women^11^ ^,^ ^16^, with records of higher prevalence of consumption or higher risk of developing dependency on nicotine among women than men^14^, at least in the younger population groups. The markets of alcohol and other drugs were spaces also conquered by women. The latest data, suggesting a changing in the epidemiological profile, contradicts the belief that women would be more protected from appeals of these markets and the risks that result by the consumption of psychoactive substances due to biological or sex related conditions. These women form a group of consumers with historically repressed access to these markets and its products, but who are now faced with another condition^h^.

We were not able to check, in this analysis, if the differences between hospitalization rates according to sex correspond to prevalence estimates of consumer behaviors also according to sex, since hospitalization rates for type of substance are not available. Moreover, modification in this indicator would depend on the modification of the profile of beds offer for the public network and information of changes of this type in the period is not available, although it may have occurred.

The profile of offer is a strong limiter of the behavior of the rates here examined, but it was exactly among women that expansion of hospitalization rates during the period occurred, albeit discreet. The variation was only 3.0%, but detectable. This finding supports what claim the reviewed authors, suggesting fluctuation of rates among women in the direction of what occurs among men^11^ ^,^ ^16^. This article also does not analyze repressed demands for hospitalization, which makes it impossible to say that the growth in demand for this type of care (people requiring this service) has been, in the period studied, the same as noted for hospitalization rates. The search for care may have grown proportionally more than the occupation of available beds. Detectable change in the profile of the occupation can reflect, therefore, qualification of health networks with enhanced entry and redistribution of the beds available among those who need it^8^.

The hospitalization rates for disorders by the use of psychoactive substances showed no statistically significant variation neither among men, nor to the set of hospitalized people. The data indicates stagnation – neither decrease, as expected, nor expansion – of the psychiatric hospitalizations for these diagnoses. The maintenance of hospitalization rates may be related to increased prevalence of psychoactive substances consumption by the population among the different age categories and by the spread of crack consumption that before was not enough to show up in surveys, but now begins to be observed. Crack causes serious damage to health and the social environment in which the individual is inserted, in addition to induce high levels of craving among users in the early days of abstinence^7^ ^,^ ^10^. This might be encouraging the appointment of hospital treatment for anyone who makes use of this substance. Stagnation may be reflecting, therefore, a balance between the pressure for increased demand and the expansion of the capacity for implementation of CAPS and local health networks.

The substitutive care model promoted in the Country, which favors care outside the hospital proposes the reduction of the importance of the hospital in the system as a whole. In practice, the hospitalization rates for 100,000 inhabitants per year or the average time of stay in hospital bed would be expected to reduce, or both. Thus, a small growth, only among women, would be pointing to a good capacity of the public network services organized in the state of Santa Catarina, Brazil, to absorb the impact of the expansion of the crack market and other substances in the Country and in the state. We did not consider in the analysis, however, the levels of occupancy of beds offered in the public system. Therefore, it is not possible to determine whether the limitation of the fluctuation of hospitalization rates does not stem from the exhaustion of the offer of beds. If the occupancy of beds is complete, the effects of the reorganization of the health care network will only be perceived when they are very intense.

The stagnation here found is similar to the results of the study by Candiago and Abreu^4^ (2007), also conducted in the South of Brazil (but with data from the state of Rio Grande do Sul) and with similar approach. In both, no significant change was observed in the total hospitalization rates throughout the studied time series, from 2000 to 2004. The implementation of community-based services in other countries have not determined the extinction of need for hospital-based services^15^ ^-^ ^19^. In the United Kingdom and several other countries, even with evidence-based public policies, beliefs persist concerning alcohol and other drugs that slow down or limit public policy transformations^11^ ^,^ ^16^.

The analysis plan developed here also does not contemplate the possibility of masked lengthy stays, as described in the phenomenon of the revolving doors^6^, in which the readmission of the same individual after short periods of stay outside the hospital raises the hospitalization rates, but maintains the reduction indicators of average time of hospitalization. This is not much different from the formal long stays, except for its presentation in statistical terms^5^ ^,^ ^9^. If this occurs, the reduction of the average time of hospitalization would be accompanied by increased estimated hospitalization rates. This is not the case of the findings here examined, but we cannot rule out that these readmissions occur. In addition, the fact that the Poisson regression analysis does not correct the serial autocorrelation that affects the time series of population measures (as the hospitalization rates) is a limitation of this study.

The interest in these data is due to the guideline policy that states funds invested in hospitalizations are reversed for the maintenance and expansion of substitute services on the psychiatric reform^13^. It is necessary to pay attention to the fact that the expansion of access of the population to mental health services can lead to the expansion of demand in all levels of care^17^ ^,^ ^22^. With this, it may be necessary to expand the structure and its technical capacity, also at the hospital level. The Brazilian reform provides for the support of psychiatric beds in general hospitals, but financing the expansion of the network of substitute services needs to be supported by a budget that does not depend on the replacement of hospital services.

The articulation of local networks in the state of Santa Catarina, Brazil, may have contributed, in the studied period, for the hospitalization rates to not have grown. Challenges remain, as posthospital follow-up services arrangements that, in fact, prevent the readmission of people in hospital care, in addition to effective integration between the different levels of attention to prevent severity levels that require this type of care^2^ ^,^ ^20^. However, estimates indicate that the management of services in the area should not count exclusively with the displacement of hospital-level features for deployment of substitute services. The expansion of local health networks, so far, does not seem to make hospital beds become obsolete in the near future.

The data source of this study was the DATASUS, a public information system on the public health system. Therefore, it is a limitation of this study not to consider information about additional network services, offered by private institutions not affiliated to SUS. In the event of a saturation of the beds offered on the public network or concurrent expansion of the number of people covered by health plans, or yet by increasing the capacity of direct costing in private services, the population may have sought care outside the public health service^21^.

Regarding the time of occupancy of hospital beds, there was no statistically significant difference over the period, a relevant fact if we consider that some reduction in the average time of hospitalization would be expected, because throughout this time series, there was expansion of the pharmacological and non-pharmacological therapies repertoire available and employed by professionals of the area^12^ ^,^ ^19^. With the expansion and qualification of local networks of services, the expectation of immediate and effective follow-up is also created, with lower pressure in the sense of the stay of the person under care in the hospital environment^21^.

It seems important to reassess the political processes and organizational arrangements that have occurred so far, aiming at the consolidation of public policies that guarantee structures for the prevention and treatment of disorders related to the consumption of psychoactive substances in non-hospital equipment. Otherwise, these indicators may never be reduced. It is important to emphasize that the data described here do not evaluate the quality and expanding of the network of substitute care to hospitalization^5^ ^,^ ^9^ ^,^ ^17^. We did not consider variables related to the services or reflections on quality. The data source of this study also does not allow for distinction, or inclusion in the analysis model, of any other variable related to the characteristics of individuals who are hospitalized, not even the identification of readmissions. This should be considered as a limitation, typical of ecological studies.

Studies with similar approach, involving inpatient care by the other mental disorders in the state of Santa Catarina, Brazil, in addition to the monitoring of all these data in other states and in the Country, need to be developed, so that a comprehensive picture of this scenario may be drawn.

## Footnotes

a Ministério da Saúde. Portaria/GM nº 336 de 19 de fevereiro de 2002. Estabelece CAPS I, CAPS II, CAPS III, CAPS i II e CAPS ad II. Brasília (DF); 2002. Available from: http://www.saude.mg.gov.br/images/documentos/Portaria_336.pdf

b Ministério da Saúde , Secretaria de Atenção à Saúde, Departamento de Ações Programáticas Estratégicas. Saúde Mental no SUS: os centros de atenção psicossocial. Brasília (DF); 2004. (Série F. Comunicação e educação em saúde).

c Brasil. Lei nº 10.216, de 6 de abril de 2001. Dispõe sobre a proteção e os direitos das pessoas portadoras de transtornos mentais e redireciona o modelo assistencial em saúde mental. *Diario Oficial Uniao.* 9 abr. 2001.

d Ministério da Saúde, Secretaria Executiva, Coordenação Nacional de DST/Aids. A política do Ministério da Saúde para atenção integral a usuários de álcool e outras drogas. Brasília (DF); 2004. (Série B. Textos básicos de saúde).

e Carlini EA, Galduróz JC, Noto AR, Carlini CM, Oliveira LG, Nappo AS, et al. II Levantamento domiciliar sobre o uso de drogas psicotrópicas no Brasil: estudo envolvendo as 108 maiores cidades do país – 2005. São Paulo (SP): UNIFESP, CEBRID; 2006.

f Carlini EA, Noto AR, Sanchez ZM, Carlini CMA, Locatelli DP, Abeid LR, et al. VI Levantamento nacional sobre o consumo de drogas psicotrópicas entre estudantes do ensino fundamental e médio das redes públicas e privadas de ensino nas 27 capitais brasileiras – 2010. São Paulo: CEBRID; Brasília (DF): SENAD; 2010.

g Organização Mundial da Saúde. Classificação Estatística Internacional de Doenças e Problemas Relacionados à Saúde: décima revisão (CID-10). São Paulo (SP): EDUSP/ Centro Colaborador da OMS para Classificação de Doenças e Problemas Relacionados à Saúde; 1993.

h Murthy P. Women and drug use in India: substance, women, and high-risk assessment study. New Delhi: United Nations Office on Drugs and Crime, Regional Office for South Asia; 2008. Available from: https://www.unodc.org/documents/southasia/reports/UNODC_Book_Women_and_Drug_Use_in_India_2008.pdf
